# The Associations of Chronotype and Shift Work With Rheumatoid Arthritis

**DOI:** 10.1177/07487304231179595

**Published:** 2023-06-29

**Authors:** Thomas Butler, J Robert Maidstone, K Martin Rutter, T John McLaughlin, W David Ray, E Julie Gibbs

**Affiliations:** * Centre for Biological Timing, Faculty of Biology, Medicine and Health, The University of Manchester, Manchester, UK; † Manchester Academic Health Science Centre, The University of Manchester, Manchester, UK; ‡ NIHR Oxford Biomedical Research Centre, John Radcliffe Hospital, Oxford, UK; § Oxford Centre for Diabetes, Endocrinology and Metabolism, University of Oxford, Oxford, UK; || Diabetes, Endocrinology and Metabolism Centre, Manchester University NHS Foundation Trust, Manchester, UK; ¶ Department of Gastroenterology, Salford Royal NHS Foundation Trust, Salford, UK

**Keywords:** circadian, shift work, rheumatoid arthritis, chronotype, epidemiology

## Abstract

The circadian clock regulates multiple aspects of human physiology including immunity. People have a circadian preference termed chronotype. Those with an evening preference may be better suited to shift work, but also carry higher risk of adverse health. Shift work leads to misalignment of circadian rhythms and is associated with increased risk of inflammatory disease such as asthma and cancer. Here, we investigate the association between chronotype, shift work, and rheumatoid arthritis (RA). The associations between exposures of shift work and chronotype on risk of RA were studied in up to 444,210 U.K. Biobank participants. Multivariable logistic regression models were adjusted for covariates: age, sex, ethnicity, alcohol intake, smoking history, Townsend Deprivation Index (TDI), sleep duration, length of working week, and body mass index (BMI). After adjusting for covariates, individuals with a morning chronotype had lower odds of having rheumatoid arthritis (RA; odds ratio [OR]: 0.93, 95% confidence interval [CI]: 0.88-0.99) when compared to intermediate chronotypes. The association between morning chronotype and RA persisted with a more stringent RA case definition (covariate-adjusted OR: 0.89, 95% CI: 0.81-0.97). When adjusted for age, sex, ethnicity, and TDI, shift workers had higher odds of RA (OR: 1.22, 95% CI: 1.1-1.36) compared to day workers that attenuated to the null after further covariate adjustment (OR: 1.1, 95% CI: 0.98-1.22). Morning chronotypes working permanent night shifts had significantly higher odds of RA compared to day workers (OR: 1.89, 95% CI: 1.19-2.99). These data point to a role for circadian rhythms in RA pathogenesis. Further studies are required to determine the mechanisms underlying this association and understand the potential impact of shift work on chronic inflammatory disease and its mediating factors.

Rheumatoid arthritis (RA) is an increasingly prevalent autoimmune chronic inflammatory disease primarily affecting the joints and significantly impacting morbidity and premature mortality (Safiri et al., 2019; Lassere et al., 2013). There is diurnal variation in symptoms of RA, with exacerbations of joint pain and stiffness in the morning, aligned to rhythmic expression of pro-inflammatory serum cytokines such as interleukin 6 (IL-6), highlighting the role of circadian rhythms in RA inflammation (Gibbs and Ray, 2013; Perry et al., 2009; Arvidson et al., 1994).

Circadian rhythms last 24 h and allow humans to distribute physiological processes across the day, using a molecular clock mechanism to keep time, and environmental stimuli such as light:dark cycles to set the time. Individuals have their own circadian phase preference, termed chronotype. While this is a continuum, people are often categorized into morning (early birds) or evening (night owls) chronotypes. Evening chronotype is associated with lower levels of health and performance (Facer-Childs et al., 2019). Our recent systematic review covered the potential association of evening chronotypes with increased disease severity across multiple immune-mediated inflammatory diseases (Butler et al., 2022).

Human circadian rhythms are coordinated by the suprachiasmatic nucleus in the hypothalamus, but peripheral clocks exist in nearly all tissues and cell types, including multiple components of the immune system. Disruption of circadian rhythms has been implicated in the pathogenesis of multiple immune-mediated inflammatory diseases including psoriasis (Luengas-Martinez et al., 2022), multiple sclerosis (Gustavsen et al., 2016), and autoimmune thyroid disease (Magrini et al., 2006). Synchronization of these clocks, and thus temporal alignment of rhythmic physiology are important for health. Misalignment between the circadian timing system and daily rhythmic behavior, including sleep-wake cycles and feeding-fasting cycles (circadian misalignment), a potential consequence of shift work or jet lag, can have significant impact on health. Shift work is becoming increasingly common worldwide, with 10%-20% of workers in most countries undertaking night shifts (Eurofound and International Labour Organization, 2019). Circadian misalignment as a result of night shift work impacts circadian processes supporting healthy immune function (Depner et al., 2018; Kervezee et al., 2018), and population data show that shift workers have a significantly higher odds of conditions with inflammatory components such as asthma (Maidstone et al., 2021b) and Covid-19 (Maidstone et al., 2021a), compared to day workers who do not work shifts. These associations persist after adjustment of multiple common covariates (Maidstone et al., 2021b, 2021a).

To explore the role of circadian rhythm disruption in individuals with RA, we assessed how strongly shift work (representing circadian misalignment) and chronotype are related to the presence of RA in U.K. Biobank participants. We hypothesized that both shift workers and evening chronotypes have an increased risk of RA. As incidence of both shift work and RA increases globally (Safiri et al., 2019), these data could have important public health implications, such as the use of circadian profiling to risk stratify shift workers.

## Methods

Between 2007 and 2010, the U.K. Biobank recruited 502,540 National Health Service-registered participants aged 40-69 years old (Sudlow et al., 2015). Baseline assessments conducted at 22 U.K. study centers collected data on lifestyle, medical history, occupation, and work patterns.

### Exposures

- *Chronotype*: Self-reported chronotype data were collected at baseline in U.K. biobank participants (*n* = 444,210) by asking: “do you consider yourself to be . . . (a) “definitely a ‘morning’ person?” or (b) “definitely an ‘evening’ person?” or (c) “more a ‘morning’ than ‘evening’ person?” or (d) “more an ‘evening’ than ‘morning’ person?”. People who responded with (a) were defined as ‘morning chronotypes’ and those who responded with (b) were defined as ‘evening chronotypes’. An ‘intermediate chronotype’ group was formed by combining responses (c) and (d). Individuals answering “do not know” (*n* = 4032) were excluded from analysis.
- *Shift work*: Shift work data were collected at baseline in U.K. Biobank participants. Shift work analysis was restricted to participants in paid employment or self-employment at baseline (*n* = 286,825, age range: 37-72 years). Shift workers were sub-categorised as working permanent night shifts or working irregular shifts, which included shift workers who never, rarely or sometimes worked night shifts.

#### Outcome

Cases of RA included patients with self-reported, doctor-diagnosed RA during baseline assessments (code 20002) or patients with International Classification of Diseases (ICD)-9/ICD-10 codes for RA diagnosis (ICD-10 codes: M05, M06 and ICD-9 code: 7140), which represent diagnoses recorded during secondary care stays prior to the U.K. Biobank baseline assessment. This identified 5,659 cases (1.3% of all U.K. Biobank participants), of which 3,537 were included in the chronotype analysis (2,122 participants answered “do not know” or “prefer not to answer”) and 2,201 were included in the shift work analysis (3,458 participants were not working at time of data collection). Sub-group analysis to increase RA case specificity included patients with self-reported, doctor-diagnosed RA who, in addition reported either a prescribed medication to treat RA (**Supplementary Table 1**) or had an ICD code for RA (shift work cohort: 1,060 cases and chronotype cohort: 2,827 cases). Unfortunately, duration of RA diagnosis and duration of medication prescription were not available.

### Statistical Analysis

Descriptive data are presented as mean (standard deviations [SDs]) or percentages unless otherwise stated. Multivariable-adjusted logistic regression models were used to generate ORs and 95% asymptotic confidence intervals (CIs) to model the relationship between shift work and chronotype with prevalent RA. Models were serially adjusted for the following covariates, selected based on differences in participant characteristics which were captured at baseline assessments (Tables 1 and 2): model 1: age, sex, ethnicity and Townsend Deprivation Index (TDI) (Townsend et al., 1988); model 2: model 1 covariates plus sleep duration, alcohol intake, smoking status, and length of working week; model 3: model 2 covariates plus BMI. The TDI is a measure of material deprivation calculated for a geographical region of the U.K. using four variables obtained from U.K. Census data (unemployment, overcrowding, non-car ownership, and non-home ownership). *Z* scores are calculated for each variable and then added to produce a Townsend deprivation score, which is used as a proxy measure for socioeconomic status. A score above zero indicates a deprived area and a score below zero indicates a more affluent area (Foster et al., 2018). TDI was chosen as a covariate for all three models as lower socioeconomic status has been identified as a risk factor for developing RA, and is associated with worse functional status in individuals with RA (Schafer and Keysser, 2022; Yang et al., 2018; Izadi et al., 2021). The impact of BMI was assessed in model 3 due to its potential involvement in the causal pathway between shift work and RA. Work by others has shown increased BMI in participants in the U.K. Biobank reporting RA compared to participants without RA (Siebert et al., 2016). Further details on each covariate can be found in **Supplementary Table 2.** Analysis was performed using statistical programming software R (version 3.6.2). R packages used include flextable (0.5.6), lubridate (1.7.4), magrittr (1.5), officer (0.3.6), patchwork (1.0.0), RColorBrewer (1.1-2), tidyverse (1.3.0), and ukbtools (0.11.0). In our analyses, we applied 5% significance levels.

## Results

### Chronotype

The characteristics of participants providing chronotype data are detailed in Table 1. When compared to intermediate chronotypes, morning chronotypes were less likely to be smokers and consumed less alcohol (but had similar BMI) and were less likely to be in paid full-time employment. Compared to intermediate chronotypes, evening chronotypes were younger, more likely to be male, smokers, with increased alcohol consumption, and more likely to be permanent night shift workers.

**Figure 1.**
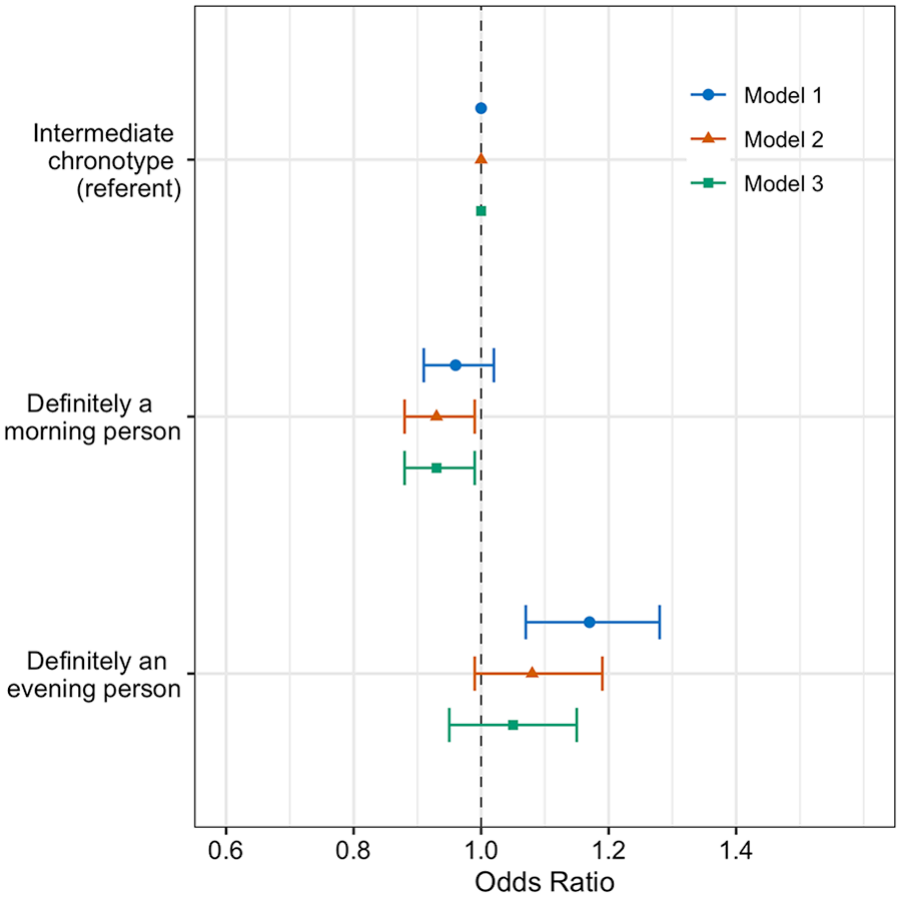
Adjusted odds ratios of rheumatoid arthritis by chronotype (*n* = 440,178). Forest plots of adjusted OR and 95% CIs for RA by chronotype, with intermediate chronotypes as a referent population. Three multivariate logistic regression models were used. Model 1 (circle): age, sex, ethnicity, and Townsend deprivation index; model 2 (triangle): sleep duration, alcohol intake, smoking status and length of working week, plus model 1 covariates; model 3 (square): BMI in addition to model 2’s covariates. Abbreviation: BMI = body mass index.

U.K. Biobank participants with an extreme morning chronotype did not have lower odds of RA, compared to intermediate chronotypes in model 1 (OR: 0.96, 95% CI: 0.91-1.02), but on further covariate adjustment, demonstrated lower odds of RA in model 2 (OR: 0.93, 95% CI: 0.88-0.99) and model 3 (OR: 0.93, 95% CI: 0.88-0.99), compared to intermediate chronotypes (Figure 1).

**Table 1.** Participant characteristics by chronotype (*N* = 440,178).

	Chronotype		
	Intermediate Chronotype	Definitely a Morning Person	Definitely an Evening Person
N	281,161	119,344	39,673
Age (years)	56.9 (8.1)	57.94 (7.8)	55.61 (8.3)
Sex (% male)	44.3	43.5	46.6
BMI (kg/m^2^)	27.3 (4.7)	27.6 (4.9)	28.0 (5.2)
Smoker (%)			
Never	54.4	56.9	45.5
Previous	35.0	33.8	35.4
Current	10.3	8.9	18.8
Smoking pack-years	6.9 (14.6)	6.8 (15.2)	10.6 (18.2)
Drink alcohol daily (%)	20.7	18.4	22.8
Sleep duration (h)	7.2 (1.2)	7.0 (1.3)	7.1 (1.4)
Ethnicity (%)			
White British	89.6	87.1	85.0
White other	5.7	6.0	8.6
Mixed	0.5	0.6	0.9
Asian	1.5	2.8	1.8
Black	1.3	2.0	1.7
Chinese	0.3	0.3	0.5
Other	5.7	6.0	8.6
Length of working week (hours)	20.2 (19.8)	19.5 (20.2)	21.1 (20.2)
Townsend deprivation index^a^	−2.3 (−3.7 to 0.3)	−2.1 (−3.6 to 0.7)	−1.7 (−3.4 to 1.2)
Work pattern (%)			
Day workers	48.3	45.3	46.7
Irregular shift work	8.3	8.5	9.1
Permanent night shift work	1.2	1.1	3.0
Not in paid full time employment or self-employed	42.2	45.0	41.2

Abbreviation: BMI = body mass index. Unless otherwise stated data are mean (SD) or percentages.

a Townsend deprivation index is a measure of socioeconomic deprivation, and expressed as median (interquartile range), where higher scores correlate with greater deprivation. See **Supplementary Table 2** for further information.

In the sub-group with more stringent RA case definition of self-reported, doctor-diagnosed RA who, in addition were either prescribed RA medication or had an RA ICD code, significant associations between morning chronotype and a lower likelihood of RA were observed in all three models: model 1 (OR: 0.91, 95% CI: 0.84-0.99), model 2 (OR: 0.89, 95% CI: 0.81-0.97), and model 3 (OR: 0.89, 95% CI: 0.81-0.97) when compared to intermediate chronotypes (**Supplementary Figure 1**).

Extreme evening chronotypes had a higher odds of RA (OR: 1.17, 95% CI: 1.07-1.28) when compared to intermediate chronotypes in the minimally adjusted model (model 1). However, the association became non-significant in model 2 (OR: 1.08, 95% CI: 0.99-1.28) and model 3 (OR: 1.05, 95% CI: 0.95-1.15) (Figure 1).

In the sub-group analysis with more stringent RA case definition, associations between evening chronotype and RA were significant compared to the intermediate chronotype referent group in model 1 (OR: 1.15, 95% CI: 1.01-1.30), but not in model 2 (OR: 1.05, 95% CI: 0.92-1.19) or model 3 (OR: 1.04, 95% CI: 0.91-1.18) (**Supplementary Figure 1**).

### Shift Work

Of participants in paid employment or self-employment, 83% were day-only workers and the remaining 17% worked shifts, of which 51% included night shifts. Compared to day workers, shift workers were more likely to be male, smokers, work longer weeks and drink less alcohol (Table 2). Night shift workers were more likely to be evening chronotypes.

**Table 2.** Participant characteristics by current shift work exposure (*N* = 286,825).

	Current Work Schedule		
	Day workers	Irregular Shift Work Schedule Including Nights	Permanent Night Shift Work
N	236,897	42,786	7142
Age (years)	53.4 (7.1)	52.46 (7)	51.5 (6.9)
Sex (% male)	46.6	53.87	61.4
BMI (kg/m^2^)	27.1 (4.7)	27.97 (4.96)	28.5 (4.9)
Smoker (%)			
Never	58.1	53.3	52.0
Previous	31.9	31.43	30.0
Current	9.8	14.86	17.7
Smoking pack-years	5.4 (12.2)	7.6 (14.8)	9.0 (16.4)
Daily alcohol intake (%)	20.5	16.5	10.2
Sleep duration (h)	7.1 (1.0)	6.91 (1.26)	6.7 (1.5)
Morning chronotype (%)	23.3	24.37	19.2
Intermediate chronotype (%)	58.6	55.8	49.4
Evening chronotype (%)	8.0	8.7	16.9
Ethnicity			
White British	88.5	81.8	81.0
White other	6.5	7.1	6.0
Mixed	0.7	0.9	0.9
Asian	1.7	3.7	3.4
Black	1.4	3.6	5.5
Chinese	0.3	0.5	0.7
Other	6.5	7.0	6.0
Townsend deprivation index^a^	−2.2 (−3.7 to 0.2)	−1.3 (−3.2 to 1.7)	−1.0 (−3.0 to 2.1)

Abbreviation: BMI = body mass index. Unless otherwise stated data are mean (SD) or percentages.

a Townsend deprivation index is expressed as median (interquartile range).

**Figure 2.**
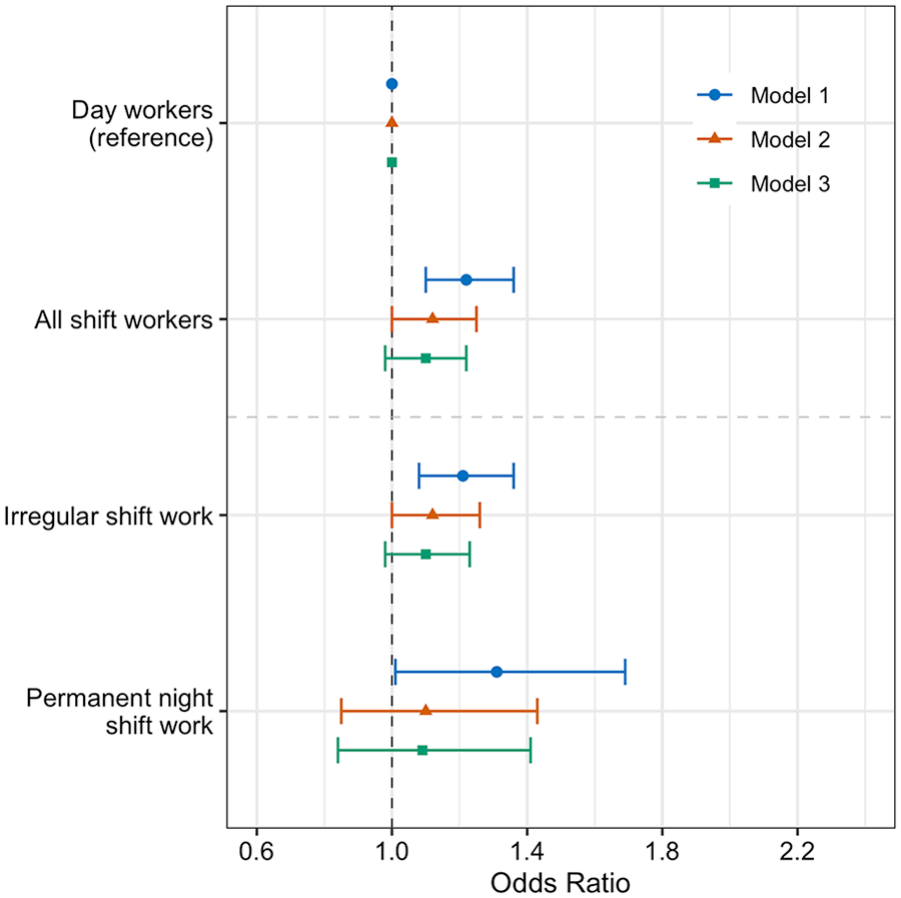
Adjusted odds ratios of rheumatoid arthritis in shift workers (*n* = 286,825). Forest plots of adjusted OR and 95% CIs for RA in all shift workers and shift workers stratified by current shift pattern, with day workers as a referent population. Three multivariate logistic regression models were used. Model 1 (circle): age, sex, ethnicity and Townsend deprivation index; model 2 (triangle): sleep duration, alcohol intake, smoking status and length of working week, plus model 1 covariates; model 3 (square): BMI in addition to model 2’s covariates. Abbreviations: OR = odds ratio; CI = confidence interval; RA = rheumatoid arthritis; BMI = body mass index.

In model 1, people undertaking any shift work had higher odds of having RA (OR: 1.22, 95% CI: 1.1-1.36) (Figure 2). Further covariate adjustment in model 2 attenuated this association (OR: 1.12, 95% CI: 1.0-1.25), *p* < 0.0001), with attenuation to the null in model 3 (OR: 1.1, 95% CI: 0.98-1.22).

Irregular shift patterns (OR: 1.21, 95% CI: 1.08-1.36) and permanent night shifts (OR: 1.31, 95% CI: 1.01-1.69), were both linked to a higher likelihood of RA, when compared to day workers in model 1 (Figure 2). However, for irregular shift workers, further adjustment for covariates in model 2 (OR: 1.12, 95% CI: 1.0-1.26) and model 3 (OR: 1.1, 95% CI: 0.98-1.23) attenuated the association, compared to day workers. Similarly, the odds of having RA in permanent night shift workers was attenuated to the null in model 2 (OR: 1.1, 95% CI: 0.85-1.43) and 3 (OR: 1.09, 95% CI: 0.84-1.41), compared to day workers.

In the sub-group analysis with more stringent RA case definition, irregular shift work (143 cases) and permanent night shift work (25 cases) were not significantly related to odds of RA, compared to day workers (**Supplementary Table 3**).

### Interaction Between Shift Work and Chronotype

To understand the influence of chronotype on the association between shift work and rheumatoid arthritis, shift workers were stratified by chronotype. (Table 3). In morning chronotypes, the odds of RA were significantly increased in permanent night shift workers, compared to day workers (OR: 1.89, 95% CI: 1.19-2.99) (Table 3). In participants with an intermediate chronotype, the odds of RA were significantly increased in irregular shift workers, compared to day workers (OR: 1.17, 95% CI: 1.01-1.37) (Table 3). In our model, an interaction between shift work schedule and chronotype was found (*p*_interaction_ = 0.02) by comparing against a null model without an interaction term. This suggests that chronotype modifies the effect of shift work on RA prevalence. To complement this approach, the influence of shift work on the association between chronotype and RA was examined. Odds of RA were significantly increased in morning chronotypes (OR: 2.16, 95% CI: 1.16-4.03) and evening chronotypes (OR: 2.16, 95% CI: 1.12-4.16) working permanent night shifts, compared to intermediate chronotypes (**Supplementary Table 4**).

**Table 3.** Adjusted odds (95% CI) of RA (ICD code or self-reported) by shift work schedule stratified by chronotype.

Current Work Schedule	Odds (95% CI)		
	Definite Morning Chronotype Odds (95% CI) (*N* = 65,586)	Intermediate Chronotype Odds (95% CI) (*N* = 162,461)	Definite Evening Chronotype Odds (95% CI) (*N* = 23,323)
Day workers	1	1	1
Irregular shift work	1.13 (0.89-1.43)	1.17 (1.01-1.37)	0.83 (0.54-1.28)
Permanent night shift work	1.89 (1.19-2.99)	0.79 (0.51-1.22)	1.59 (0.94-2.68)
***P*_interaction_**	0.02		

Abbreviations: CI = confidence interval; RA = rheumatoid arthritis; ICD = International Classification of Diseases.

## Discussion

This study analyzed data from over 440,000 U.K. Biobank participants and demonstrates lower adjusted odds of RA in individuals with a morning chronotype compared to an intermediate chronotype, which persists when a more stringent RA case definition is used. In addition, this study demonstrates an association between shift work and prevalent RA which is attenuated on covariate adjustment. There is a significant interaction between shift work and chronotype in RA risk. In morning chronotypes, permanent night shift work is associated with significantly higher odds of RA, compared to day workers.

Our results are contrary to a recent paper, where authors used the Munich Chronotype Questionnaire to report an earlier mid-point of sleep in 121 RA cases from the Netherlands, compared to healthy controls (Habers et al., 2021). Our study involved a much larger sample size and adjustment for multiple covariates, but our case definition specificity may be lower due to reliance on a combination of doctor-diagnosed RA and ICD codes, rather than rheumatologist-diagnosed RA. In our analysis, morning, compared to intermediate chronoty pe is linked to risk factors associated with higher RA prevalence such as lower daily alcohol intake, shorter sleep duration and shorter working week, and therefore when these factors are accounted for in model 2, the odds of RA associated with morning chronotype is lowered and the association becomes statistically significant. This pattern persists in model 3. Interaction analysis of morning chronotypes identified higher odds of RA in permanent night shift workers compared to day workers. This suggests the lower risk of RA in morning chronotypes is attenuated in morning chronotypes who undertake permanent shift work. Further work is required to understand whether patients with RA have a chronotype shaped by rhythmicity in symptoms, against a morning preference when joints are most painful. In addition, the potential mediator role of these covariates, such as shorter sleep duration should be explored in future research, in order to better understand specific targets for intervention.

Our study demonstrated higher adjusted odds of RA in shift workers, including those who never or rarely work night shifts and permanent shift workers, when compared to day workers. However, this association attenuated to the null in models 2 and 3. These results would be in keeping with sleep duration, smoking and/or BMI being confounders, and/or mediators of the relationship between shift work and RA. Further work will be required to elucidate these potential mechanistic links. Shift workers constantly expose themselves to the risk of circadian misalignment and the systemic consequences of desynchrony between internal clocks and the external environment designates shift work a risk factor for chronic diseases such as asthma, diabetes, and cancer (Sletten et al., 2020; Maidstone et al., 2021b; Vetter et al., 2018). Our data provide evidence that RA should be considered in this list.

This study builds on two previous smaller studies analyzing shift work and RA. In a Finnish population of public sector workers, women working night shifts had a higher risk of incident RA when compared to day workers (Puttonen et al., 2010). In a Swedish case-control study, rotating shift work was associated with a higher risk of incident seropositive RA, whereas permanent night shift work conferred a protective effect (Hedström et al., 2017).

A strength of our study is the large sample of RA cases from the U.K. Biobank, which also contains data to enable robust adjustment for covariates that impact chronotype, such as age, sex, and ethnicity (Roenneberg et al., 2019). The protective association between morningness and RA persisted through models controlling for these covariates. In patients with RA, inflammatory cytokines peak in the morning (Gibbs and Ray, 2013). Taken in context of our findings, this raises the interesting possibility that biological clocks in morning types, rather than intermediate or evening types, are more suited to regulate and control inflammatory processes that drive RA pathogenesis, thus avoiding RA development. Further profiling of morning types, including circadian-driven meal timing, activity profiles and sleep hygiene may identify protective factors that could help reduce RA risk in non-morning types and highlight potential chronotherapeutic avenues such as chronotype-driven drug timing. In our study, the trend toward higher odds of RA in evening types did not reach statistical significance. Evening chronotype has been associated with increased severity of chronic inflammatory diseases including RA, asthma, and inflammatory bowel disease (IBD), as assessed by multiple quality-of-life indices (Chakradeo et al., 2018; Kantermann et al., 2012; Butler et al., 2022). It was not possible to risk stratify RA cases by severity in this study, as data required to calculate severity scores such as disease activity score 28 (DAS28) were not available. Further work should explore the association of chronotype and RA severity to determine whether chronotype could be used as a screening tool to risk stratify patients with RA.

The U.K. Biobank has inherent limitations, for example, the participation rate was only 5% of invited people, and participants were more healthy than the general population (Fry et al., 2017). Over half of the RA cases had no recorded medication for RA, which raises the possibility of misdiagnosis. To investigate this, sub-group analysis aimed to increase specificity of RA case definition by cross-referencing self-reported, doctor-diagnosed RA cases with the presence of either an ICD-10 code for RA or presence of RA medication. While this decreased case numbers by 2,832, the lower odds of RA in extreme morning chronotypes persisted, compared to intermediate chronotypes. This gives support to the suitability of RA case definition used in the primary analysis and the main findings. RA case numbers in shift work analyses were low, with only 62 permanent night shift workers identified, which may be due to the high levels of withdrawal from paid employment seen in RA patients (Backman, 2004), impacting the power of statistical tests and increasing the possibility of type II error. In addition, this cross-sectional analysis only captures information at time of recruitment. It is possible that those identifying as day workers or day shift workers at the U.K. biobank baseline assessment may have recently changed from a prolonged period of night shift work, perhaps even following a diagnosis of RA, which may underestimate the true effect size in this study. Furthermore, we were unable to consider length of exposure to permanent shift work, and we acknowledge that the duration of exposure could be a critical factor here. Furthermore, analysis only included prevalent RA cases at U.K. Biobank enrolment.

## Conclusion

This study reports an association between morning chronotype and lower risk of RA as well as an increased adjusted odds of RA in shift workers. This adds to the mounting evidence that circadian rhythms are a key factor in RA and adds strength to the idea that circadian misalignment, through shift work or individual circadian preference through chronotype, may be important in RA development. Further studies are required to explore the mechanistic links between circadian misalignment and development, or propagation of inflammation seen in RA.

## Supplemental Material

sj-docx-1-jbr-10.1177_07487304231179595 – Supplemental material for The Associations of Chronotype and Shift Work With Rheumatoid Arthritis Supplemental material, sj-docx-1-jbr-10.1177_07487304231179595 for The Associations of Chronotype and Shift Work With Rheumatoid Arthritis by Thomas Butler, J Robert Maidstone, K Martin Rutter, T John McLaughlin, W David Ray and E Julie Gibbs in Journal of Biological Rhythms
