# Supplementary material for: The Associations of Chronotype and Shift Work With Rheumatoid Arthritis
Source: J Biol Rhythms. 2023 Jun 29;38(5):510–8. doi: 10.1177/07487304231179595 (PMC10475206; doi:10.1177/07487304231179595)
Supplement: sj-docx-1-jbr-10.1177_07487304231179595 – Supplemental material for The Associations of Chronotype and Shift Work With Rheumatoid Arthritis [file sj-docx-1-jbr-10.1177_07487304231179595.docx]

**Supplemental material: The associations of chronotype and shift work with rheumatoid arthritis**

Butler TD*^1^, Maidstone RJ^4^*, Rutter MK^1,2^, McLaughlin J^1,3^, Ray DW^4†^ and Gibbs JE^1†^

**Supplementary Table 1**

**Supplementary Table 2**

**Supplementary Table 3**

**Supplementary Table 4**

**Supplementary Figure 1**

**Supplementary Table 1.**

| Steroids |
| --- |
| Corticosteroids |
| Depomedrone |
| Triamcinolone |
| Methylprednisolone |
| Prednisolone |
| Prednisone (UK Biobank: Deltacortril enteric; Deltastab; Precortisyl; Prednesola) |
| Disease-modifying anti-rheumatic drugs (DMARDs) |
| Auranofin (UK Biobank: Ridaura) |
| Azathioprine (UK Biobank: Imuran) |
| Hydroxychloroquine (UK Biobank: Plaquenil) |
| Leflunomide (UK Biobank: Arava) |
| Methotrexate |
| Methotrexate injections |
| Myocrisin |
| Penicillamine |
| Sulfasalazine (UK Biobank: Sulazine; salazopyrin; sulphasalazine) |
| Biologics (monoclonal antibodies) |
| Abatacept |
| Adalimumab (UK Biobank: Humira injection solution) |
| Certolizumab |
| Etanercept |
| Golimumab |
| Infliximab |
| Rituximab |
| Tocilizumab |

**Supplementary Table 1. Medications for RA recorded by UK Biobank participants**

**Supplementary Table 2.**

| Covariate | Definition/units | Categories |
| --- | --- | --- |
| Age | Years | Continuous |
| Sex |  | Categorical: male; female |
| Ethnicity | ‘What is your ethnic group?’ | Categorical: White British; White other, mixed, Asian, Black, Chinese, other |
| Townsend deprivation index | A measure of material deprivation in a population. Calculated from preceding national census data. Participants assigned score corresponding to their postcode. | Continuous. Z scores about a mean of 0. Score > 0 is more deprived, score < 0 is more affluent |
| Sleep duration | ‘How many hours sleep do you get in every 24 hours (including naps)?’ | Whole number categories |
| Alcohol intake | ‘How often do you drink alcohol?’ | Categorical: daily or almost daily; 3-4 times a week; 1-2 times a week; 1-3 times a month; special occasions only; never |
| Smoking status | Current smoking status | Categorical: never; previous; current |
| Length of working week | ‘In a typical week, how many hours do you spend at work?’ | Whole number categories |
| Body mass index | Weight/height^2^ | Continuous |

**Supplementary Table 2. Description of covariates used in multiple logistic regression models in this paper**

**Supplementary Table 3.**

|  | Current work schedule | | |
| --- | --- | --- | --- |
|  | **Day workers** | **Irregular shift work** | **Permanent night shift work** |
| **Total cases (% of total sample size)** | 892 (0.38%) | 143 (0.34%) | 25 (0.35%) |
| **Total sample size** | 233,423 | 41,826 | 7,054 |
| **Model 1** | 1 | 0.99 (0.83-1.18) | 1.11 (0.75-1.66) |
| **Model 2** | 1 | 0.91 (0.76-1.09) | 0.94 (0.63-1.4) |
| **Model 3** | 1 | 0.91 (0.76-1.09) | 0.94 (0.63-1.41) |

**Supplementary Table 3. Subgroup analysis of adjusted odds (95% CI) of rheumatoid arthritis (self-reported with either medication or ICD code) in shift workers by shift work schedule (N = 282,303).**

Model 1: age, sex, ethnicity and Townsend deprivation score; model 2: sleep duration, alcohol intake, smoking status and length of working week, plus model 1 covariates; model 3: BMI in addition to model 2’s covariates.

**Supplementary Table 4.**

|  | Odds (95% CI) | | |
| --- | --- | --- | --- |
| **Current work schedule** | **Day worker**  **Odds (95% CI)**  (N= 208,430) | **Irregular shift worker**  **Odds (95% CI)**  (N= 39, 945) | **Permanent night shift worker**  **Odds (95% CI)**  (N= 5,995) |
| Intermediate chronotype | 1 | 1 | 1 |
| Definite morning chronotype | 0.91 (0.81-1.02) | 0.88 (0.68-1.13) | 2.16 (1.16-4.03) |
| Definite evening chronotype | 1.09 (0.92-1.29) | 0.75 (0.49-1.14) | 2.16 (1.12-4.16) |
| **P_interaction_** | 0.02 | | |

**Supplementary Table 4. Adjusted odds (95% CI) of RA (ICD code or self-reported) by chronotype stratified by shift work schedule.** Analysis performed with model 2 covariates. P_interaction_ represents analysis of chronotype as a potential modifier of the association between shift work and chronotype.

**Supplementary Figure 1.**

**
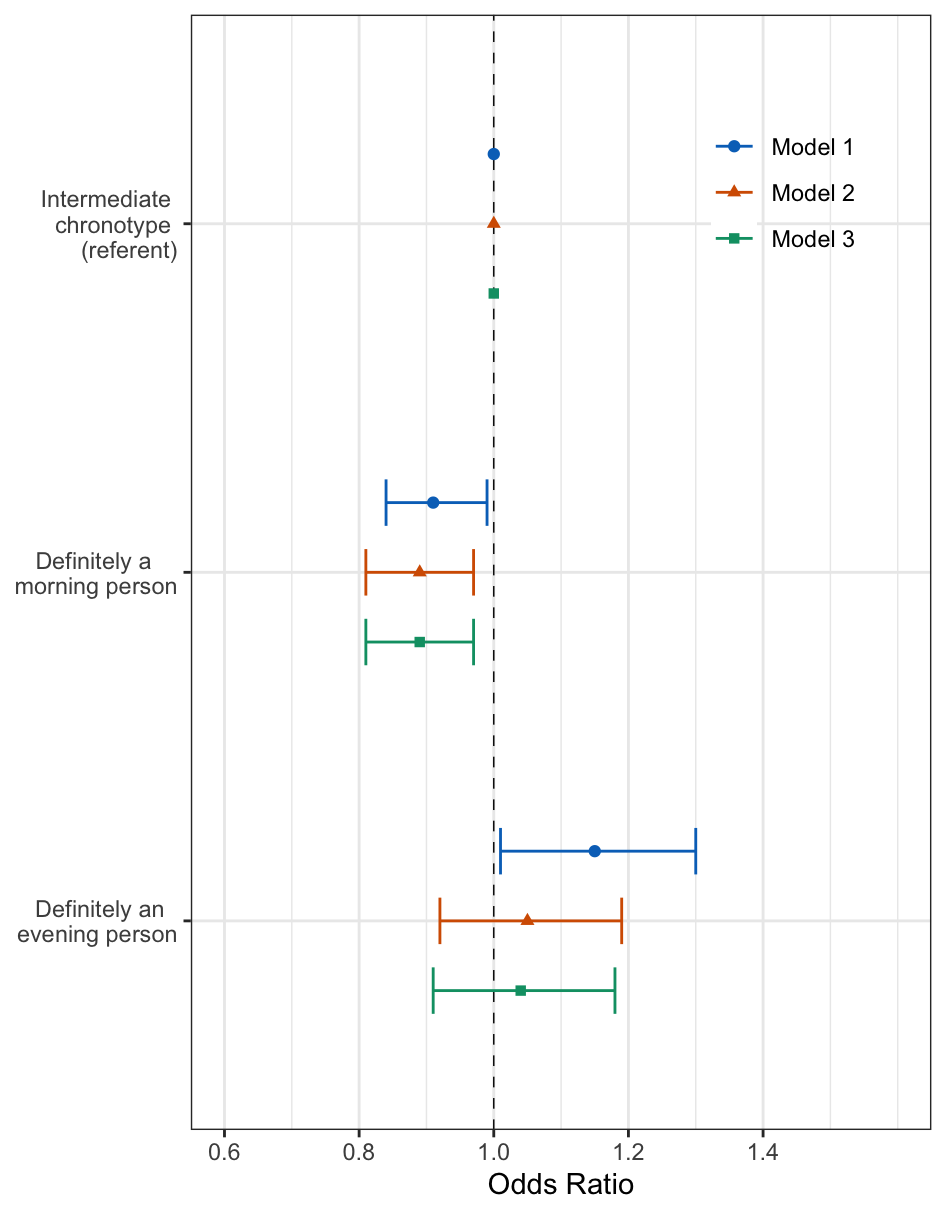
**

**Supplementary Figure 1. Subgroup analysis of adjusted odds of rheumatoid arthritis (self-reported with either medication or ICD code) by chronotype (n=440,178).**

Forest plots of adjusted OR and 95% CIs for RA by chronotype, with intermediate chronotypes as a referent population. Three multivariate logistic regression models were used. Model 1 (circle): age, sex, ethnicity and Townsend deprivation score; model 2 (triangle): sleep duration, alcohol intake, smoking status and length of working week, plus model 1 covariates; model 3 (square): BMI in addition to model 2’s covariates.
